# Mixture modeling of transcript abundance classes in natural populations

**DOI:** 10.1186/gb-2007-8-6-r98

**Published:** 2007-06-04

**Authors:** Wen-Ping Hsieh, Gisele Passador-Gurgel, Eric A Stone, Greg Gibson

**Affiliations:** 1 Department of Genetics, Gardner Hall, North Carolina State University, Raleigh, North Carolina 27695-7614, USA; 2 Department of Statistics, 825 General Building III, National Tsing Hua University, Kuang-Fu Road, Hsinchu, 30013, Taiwan; 3 Department of Statistics, and Bioinformatics Research Center, 1500 Partners II Building, 840 Main Campus Drive, North Carolina State University, Raleigh, North Carolina 27695, USA

## Abstract

**Background:**

Populations diverge in genotype and phenotype under the influence of such evolutionary processes as genetic drift, mutation accumulation, and natural selection. Because genotype maps onto phenotype by way of transcription, it is of interest to evaluate how these evolutionary factors influence the structure of variation at the level of transcription. Here, we explore the distributions of *cis*-acting and *trans*-acting factors and their relative contributions to expression of transcripts that exhibit two or more classes of abundance among individuals within populations.

**Results:**

Expression profiling using cDNA microarrays was conducted in *Drosophila melanogaster *adult female heads for 58 nearly isogenic lines from a North Carolina population and 50 from a California population. Using a mixture modeling approach, transcripts were identified that exhibit more than one mode of transcript abundance across the samples. Power studies indicate that sample sizes of 50 individuals will generally be sufficient to detect divergent transcript abundance classes. The distribution of transcript abundance classes is skewed toward low frequency minor classes, which is reminiscent of the typical skew in genotype frequencies. Similar results are observed in reported data on gene expression in human lymphoblast cell lines, in which analysis of association with linked polymorphisms implies that cis-acting single nucleotide polymorphisms make only a modest contribution to bimodal distributions of transcript abundance.

**Conclusion:**

Population surveys of gene expression may complement genetical genomics as a general approach to quantifying sources of transcriptional variation. Differential expression of transcripts among individuals is due to a complex interplay of cis-acting and *trans*-acting factors.

## Abstract 2

Expression profiling of *Drosophila melanogaster *adult female heads for 108 nearly isogenic lines from two different populations, and of CEPH lymphoblastoid lines, shows that differential expression of transcripts among individuals is due to a complex interplay of cis- and trans-acting factors.

## Background

It is well known that the structure of genetic and phenotypic variation within and between populations is affected in a complex manner by drift, migration, mutation, and selection. Because the genotype is connected to the phenotype via transcript abundance, it behooves us to attempt to ascertain the population structure of transcriptional variation as well. Although robust theory exists describing the expected distribution of genotypic variation under a variety of evolutionary scenarios [1-3], there is no theory describing the expected distribution of transcriptional variation, and neither are there many empirical data in this regard.

Numerous studies conducted in a range of species have demonstrated that transcript abundance typically exhibits moderate to high heritability [4-6]. Differential expression in the range of 1.5-fold to 2-fold between any two individuals is often seen for at least 10% of transcripts, whereas as many as one half of all transcripts may be variable in a large sample of individuals. Expression quantitative trait locus (QTL) studies demonstrate a genetic component to much of this variation that is due both to *cis*-acting and *trans*-acting factors, and frequently more than 25% of the transcriptional variance can be attributed to single regulatory QTLs (for review [7,8]). Because it is now believed that regulatory polymorphism is prevalent in eukaryotic genomes [9], it follows that there is ample opportunity for the distribution of transcript abundance to diverge between populations within a species [10,11]. The rate of divergence should be proportional to the level of variation within populations, and this observation motivates the development of quantitative measures of transcriptional variation among individuals.

Transcriptional population structure can be described using parameters that capture the mean, range, variance, and skewness of the frequency distribution of each transcript measured by microarray analysis of individuals or inbred lines. Whereas allele frequencies involve discrete entities, namely single nucleotide polymorphisms (SNPs) or indels, that can be counted and compared, transcript abundance is continuous. It is therefore subject to measurement error, and robust statistical approaches are needed to compare distributions, preferably using likelihood-based measures. It turns out that measurement of the descriptive parameters is strongly affected by experimental methods as well as analytical approaches such as normalization methods, and consequently epistemologic issues must be confronted in the description of transcriptional population structure.

To the extent that transcript abundance is strongly affected by major regulatory factors, it may also be possible to observe bimodal or even multimodal distributions. The relative weight of these modes should vary among populations as a result of divergence in allele frequency of the regulatory factors. Thus, if a promoter polymorphism that reduces transcription measurably in homozygotes is at a frequency of 0.2 in one population and 0.5 in another, then the relative abundance of the low transcript abundance class will be expected to be less than 5% in the first and as much as 25% in the second population. Depending on the degree of dominance of the effect, two or three 'transcript abundance classes' (TACs) will be detected. If the regulatory polymorphism affects the abundance or activity of a *trans*-acting factor, then the abundance of numerous target genes should be affected in parallel, resulting in 'transcriptional cliques' that exhibit correlated patterns of gene expression across a sample of individuals [6].

In this report we document the existence of TACs in a large sample of two North American populations of *Drosophila melanogaster*, as well as in previously published data on gene expression in lymphoblast cell lines from the Centre d'Etude du Polymorphisme Humain (CEPH) grandparents [12,13] (also see the CEPH website [14]). In both cases the distribution of minor TAC frequencies is observed to approximate the expected distribution of allele frequencies under an infinite sites model, because there is an excess of minor TACs with frequencies less than 10%. This observation is consistent with the hypothesis that a considerable proportion of transcriptional variation might be attributed to segregating neutral or nearly neutral alleles, but follow-up association tests in the CEPH data indicate that only a small proportion of the bimodality is actually attributable to *cis*-acting polymorphisms. Population profiling should be considered a complement to genetical genomics [8] for dissecting the quantitative genetics of gene expression.

## Results

### Transcriptional divergence between North Carolina and California populations

Population-based gene expression profiling of adult female *Drosophila *heads was performed using cDNA microarrays, as part of a study of the quantitative genetic basis for nicotine resistance in *Drosophila melanogaster *[15]. A total of 216 hybridizations were performed, with each array contrasting RNA from control and nicotine-treated flies derived from two different lines from either a North Carolinian (NC) sample of 58 lines or a Californian (CA) sample of 50 lines. A randomized loop design [16] was used with just two replicates of each line and drug treatment, one for each of the Cy3 and Cy5 fluorescent dyes. Each array contains 4,385 unique expressed sequence tag amplicons that were initially isolated by the Berkeley Drosophila Genome Project [17].

**Figure 1:**
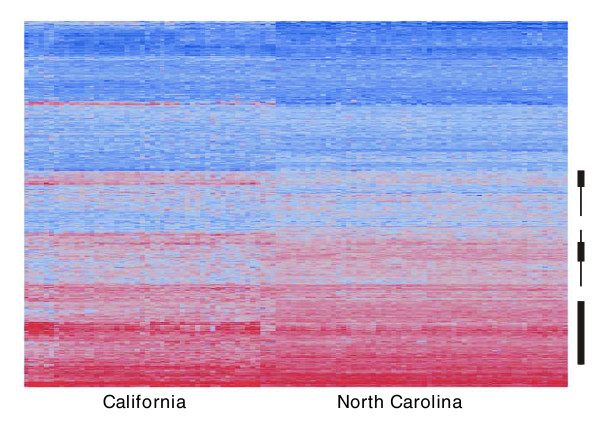
Two-way hierarchical clustering of abundance of all transcripts in NC and CA samples. The heat map indicates relatively high abundance in magenta and low abundance in blue, with each row corresponding to one gene and each column one line of flies. Thick bars to the right indicate genes that appear to differentiate the NC and CA samples, whereas the thin bars highlight genes that have polymorphic expression in both samples. CA, California; NC, North Carolina.

Following quality control and normalization (as described in Materials and methods [see below]), two-way hierarchical clustering was performed to visualize the overall structure of variation in the entire sample. In Figure 1 each row is a transcript, and each column a line of flies. Magenta signifies relatively high transcript abundance and blue low abundance. Two results are immediately obvious. First, lines from each of the two populations form two distinct clusters, due largely to hundreds of genes that apparently have different relative abundance between the NC and CA samples, many of which are indicated by thick lines to the right of the heatmap. Second, some genes are more variable among lines than others, in both populations, and some of these that cluster together are highlighted with thin vertical lines.

The apparent, striking divergence between NC and CA is almost certainly over-estimated by this analysis, because the population of origin of each line was confounded by an experimental batch effect. For reasons unrelated to this study, the NC and CA hybridizations were performed 4 months apart. In an attempt to confirm the differentiation, after the initial analysis was completed a series of hybridizations was performed contrasting lines from each population on the same microarrays. These new samples did not separate the populations cleanly, and cluster as their own group within the NC cluster, when they are analyzed together with the main dataset (data not shown). The reasons for the batch effect are unclear, because two slide printing runs and batches of enzyme were performed with each sample, and the same person (GPG) performed all of the hybridizations. It may pertain to an ozone effect or some other seasonal variable [18]. In any case, the mean differences in inferred transcript abundance across the 58 NC and 50 CA lines are not a reliable indicator of transcriptional divergence between the populations in this dataset.

**Figure 2:**
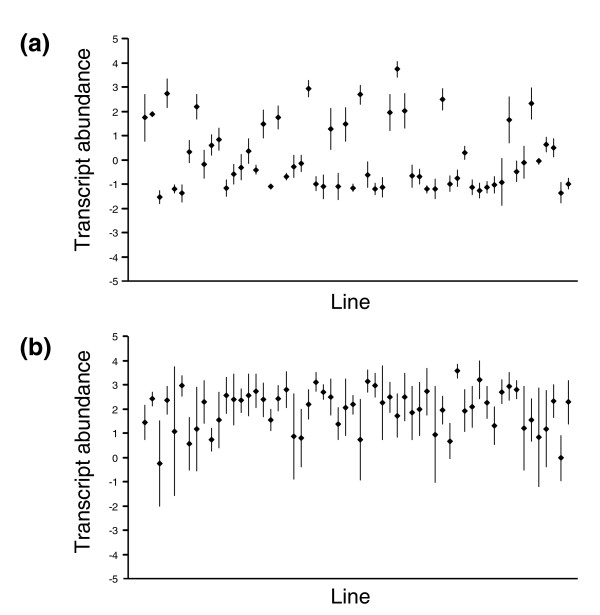
Line means for two typical transcripts across the NC sample. Each plot shows the mean relative fluorescence intensity on a log base-2 scale for the four samples (two control and two nicotine-treated) of each line in random order (± 1 standard deviation unit). **(a) **CG7843 (unknown gene that is predicted to be involved in defense/toxin response) is an example of a gene with bimodal abundance, with the minor transcript abundance class centered approximately fourfold more abundant than the average transcript on the array (relative fluorescence intensity = +2), and the major transcript abundance class (TAC) twofold less abundant than the average (relative fluorescence intensity = -1). **(b) **CG12141 (encoding Lysyl tRNA synthetase) is a gene with a single mode of transcript abundance, given the variance among and within lines.

By contrast, there are several interesting patterns of variation among lines that may be more informative indicators of transcriptional population structure. Figure 2 plots the relative fluorescence intensity, averaged across all four measurements for each NC line (that is, two dyes and two drug conditions), for one gene that exhibits strong variance among lines (Figure 2a) and for one that is fairly uniform (Figure 2b). As noted by others, the power to detect line effects in an experiment with low replication is low [4,5] but, depending on the method of normalization and the population, between 3% and 11% of the 4,385 transcripts exhibit a random line effect that is greater than the residual error in an analysis of variance (Table 1). This is likely to be an underestimate of the number of genes that exhibit significant heritability for transcription, because replicated comparison of the most extreme lines for each gene would indicate many more significant differences.

**Table 1:** Number of bimodally expressed genes

Sample	Model^a^	Line effect^b^	Bimodal^c^
NC	Raw data	297	206
	Mixed model normalization	192	324
	Loess normalization	470	304
	Both mixed and loess	188	162
CA	Raw data	285	243
	Mixed model normalization	119	409
	Loess normalization	406	319
	Both mixed and loess	114	131
Common to both CA and NC		204	69^d^

^a^'Raw data' refers to analysis directly on the log transformed raw fluorescence intensity measures, without normalization to remove array effects. 'Mixed model' refers to gene-specific models after mixed model normalization (as described in Materials and methods). 'Loess normalization' refers to analysis after loess treatment of the arrays. Note that loess increases the number of genes with significant line effects, but it reduces the number with apparent bimodality. ^b^The number of genes exhibiting greater line variation than the residual when treating the line effect as a random factor. ^c^The number of genes for which the mixture modeling indicates a greater likelihood that the distribution of transcript abundance across lines has two or more modes. ^d^The total number of genes with bimodal expression in both populations, either from the mixed (48 genes), loess (33 genes), or both modes of analysis (12 genes). CA, California, NC, North Carolina.

For most individual genes, the range and variance of transcript abundance are very similar between the two populations. Comparison of these parameters does not provide any evidence for divergence in variability between the populations. Although the mean transcript abundance for each population is often significantly different, as described above, this may be attributed to batch and normalization artifacts. A more robust approach to detecting transcriptional divergence is to define first the structure of variation within each population, focusing on the distribution of variation within the NC and CA samples considered separately.

### Mixture modeling of bimodal transcript distributions

**Figure 3:**
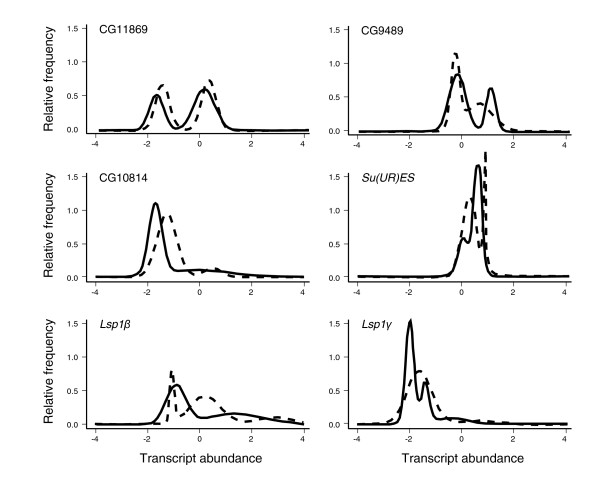
Six examples of bimodal TACs in both populations. Each plot shows the frequency distribution in the North Carolina (NC) sample (solid curve) and California (CA) sample (dashed curve). Units along the x-axis are log base-2 relative fluorescence intensity after mixed model normalization. The top two rows show transcripts with similar distributions in both populations. The bottom two rows show two transcripts with apparently different distributions in NC and California (CA), both encoding larval serum proteins. TAC, transcript abundance class.

If major effect alleles influence gene expression, then transcript abundance might be expected to split into two or more modes. Rather then asking whether the frequency distribution of abundance deviates from a single normal distribution, we employed mixture modeling [19] to evaluate whether the data are explained better by superposition of multiple distributions. This analysis was performed on each population separately to avoid confounding by the overall population/batch effects. Mclust software [20,21] was used to identify the optimal weighting of and deviation between *n *modes that maximizes the likelihood. A Bayesian Information Criterion was then employed to choose the best model with *n *= 1, 2, 3, 4, or 5 modes. Simulations assuming a single normal distribution of expression values established a false-positive rate of 4% for identification of bimodal distributions. By contrast, evaluating each population separately, we detected between 7% and 10% of transcripts as having bimodal or trimodal abundance distributions in both the NC and CA populations. Table 1 shows the number of transcripts assigned to multiple modes for population as well as combined analyses. The percentage of genes common to both populations is approximately 12% of the number in either population alone, implying significant overlap, with 48 genes at least bimodal in both the NC and CA samples following mixed model normalization, and 33 following loess normalization. Several examples of transcripts with bimodal distributions that have similar shapes in both populations are provided in Figure 3.

**Figure 4:**
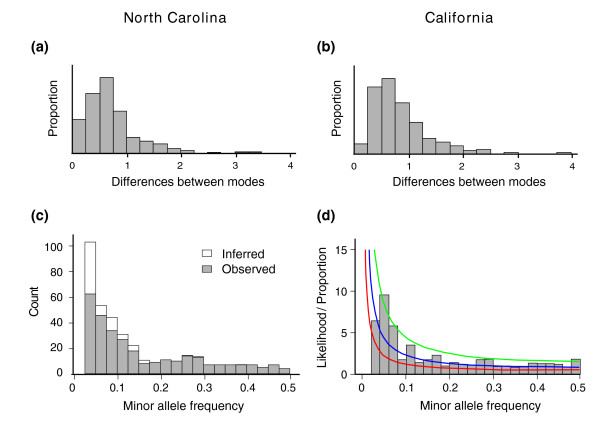
Parameters of bimodal transcription abundance classes in Drosophila by population. **(a, b) **Histograms of magnitude of differences between modes of the two transcript abundance classes (TACs), on a log base-2 scale, in North Carolina (NC) and California (CA), respectively. In both populations the median difference is between 1.5-fold and 2-fold, but a few transcripts exhibit differences as great as 16-fold. **(c) **Histograms of observed (solid bars) and inferred (open bars) minor TAC frequencies in the NC sample. **(d) **Histogram of observed distribution of minor TAC frequencies in the CA sample, relative to expected minor single nucleotide polymorphism frequencies under the Ewens sampling distribution, with the population parameter θ (that is, 4Nμ) equalling 0.05 (red line), 0.10 (blue line), or 0.20. The two curves for the most part lie within the range of expected values for *D. melanogaster *defined by the red and blue curves, although there is a slight excess of minor transcript frequencies between 5% and 10%.

Given this evidence that almost twice as many genes are expressed bimodally than expected by chance, we can assign transcripts to TACs. Figure 4 panels a and b show the distribution of differences between the means of the major and minor TACs for each transcript in the NC and CA samples respectively; panels c and d show the proportion of alleles in the minor TAC. Most TACs diverge between 1.5-fold and 4-fold, but differences as great as 16-fold are observed occasionally; these typically involve just a handful of lines in the minor TAC. There is also some suggestion that expression differences tend to be greater in the CA sample.

The distribution of minor TAC proportions is decidedly L-shaped; the majority of minor modes contain fewer than 10% of the transcript abundance measures, but there is a range of values up to equal frequency of the low and high classes. This observation is reminiscent of the distribution of genotype frequency classes known as the Ewens sampling distribution [22,23]. The most parsimonious explanation for this similarity would be that rare alleles segregating under neutrality act in *cis *to drive the observed bimodality of transcription. In Figure 4d we have superimposed the expected distribution of SNP frequencies under an infinite sites model for three different values of the population parameter *4Nμ *on the observed distributions of minor transcript abundance classes in the CA sample. The lower two curves represent expected values for *Drosophila melanogaster *[24], and the histogram of the transcript distribution lies within this range, which is consistent with this simple explanation. Unfortunately, there is no current theory by which to derive an expected distribution of TACs under alternative models of regulation. *Trans*-acting polymorphisms under some scenarios may produce a similar distribution of TACs.

In evaluating the relationship between the TAC and SNP frequency distributions, there are numerous issues of ascertainment bias that remain to be addressed. There appears to be a slight excess of minor TACs in the range of 0.05 to 0.1 in both populations, but this may be a result of a strong tendency to underestimate the number of rare TACs observed in just one or two lines, as well as failure to detect TACs with only small mean differences. We used simulations to estimate the false-negative rate for each of these two classes of error, and used those estimates to infer more realistic true distributions of TACs (see Figure 2c for the NC sample). The precise shape of these distributions is heavily influenced by error in the detection of rare TACs, and so there is little point in performing tests of goodness-of-fit between TAC and SNP distributions, but it is clear that there is a heavy skew toward an excess of rare or intermediate frequency TACs.

In *Drosophila*, the high level of polymorphism combined with a low level of linkage disequilibrium, and hence haplotype block structure, impedes association mapping using tagging SNPs [25-27]. To test whether *cis*-acting SNPs might account for TACs, we sequenced, from 43 of the NC lines, a short 1.8 kilobase (kb) gene (CG31231) that is sandwiched tightly between two other genes and that exhibits transcriptional bimodality in both populations. Three out of 16 common, independently segregating SNPs were observed to correlate with transcript abundance, one being a synonymous substitution with a rare allele frequency of 0.23 that explains 9% of the transcript abundance at *P *= 0.03 (*t*-test) on both control and nicotine diets. This SNP accounts for less than half of the bimodality of CG31231 expression and would not be detected in a genome scan for association with expression.

### Power to detect transcriptional abundance classes

**Figure 5:**
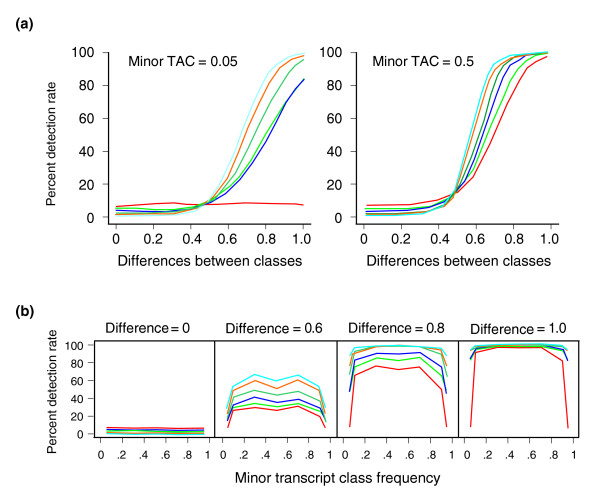
Power studies. **(a) **Percent detection rate as a function of the difference between the modes of the two transcript abundance classes, for minor transcript abundance class (TAC) frequencies of 0.05 (left) and 0.5 (right). Colors represent increasing sample size, from 30 lines (red) to 40 (blue), 50 (green), 70 (blue-green), 90 (orange), or 100 (light blue) lines. Power of 80% is obtained for 100 lines if the modes differ by more than 1.7-fold (1.75 log base-2 units), and 40 lines if they differ by more than 2-fold. Thirty lines is too few to perform this type of analysis. **(b) **Percentage detection rates as a function of minor TAC proportion, for four different values of the difference between median expression value of each class. Power drops quickly for minor TACs less than 10% of the sample, but it is fairly constant for all other relative abundances of the two classes.

Many truly multimodal distributions will appear as skewed single normal distributions. This is most likely to occur where the expression is noisy, the magnitude of expression difference between the abundance classes is small, or the frequency of the minor class is small. To investigate the effects of sample size, the magnitude of differentiation, and proportion of abundance classes on power to detect bimodal expression, Monte Carlo simulations were performed. The standard deviation of the line means was held constant at 0.2 log base-2 units (based on the average standard deviation in the *Drosophila *experiments) and 3,000 datasets were simulated. Power is estimated as the detection rate of bimodality using the mixture modeling approach. The results are presented in Figure 5.

Sample sizes of at least 50 lines appear to be quite adequate for detection of bimodality across a range of minor TAC frequencies (Figure 5a). Whereas 30 lines is insufficient for a minor proportion of 0.05, 80% detection rate is achieved for a twofold difference in magnitude between the minor and major TAC means so long as at least 50 lines are surveyed. This threshold reduces to 1.7-fold for surveys of 100 lines. For equal proportions of the two TACs, a similar power is observed irrespective of the sample size. Consequently, if at least three out of a sample of 50 or more lines are 1.7-fold differentially expressed relative to the remainder of the sample whose standard deviation is less than 1.2-fold, there is good power to detect differential expression. Clearly, satisfaction of these criteria is more likely as the quality of the microarrays improves and more replication is performed.

Furthermore, detection rates are only strongly affected when the frequency of the minor TAC drops below 10% (Figure 5b). For a 1.5-fold difference in abundance (that is, 0.6 log base-2 units), the detection rate ranges from 30% to 70% as sample size increases from 30 to 100 lines and the proportion of the minor TAC is greater than 0.1. Subsets of fewer than five lines are only assigned to a separate mode if they are at least twofold divergent from the major mode. Because about half of the observed bimodal transcript distributions have a minor TAC less than 10%, whereas two-thirds of them have a difference greater than twofold, it follows that most of the more divergent TACs are due to relatively rare alleles. Conversely, rare alleles of small effect are likely to go undetected in population surveys of expression.

Such rare alleles may still contribute to skew of normal distributions; therefore, we also examined the effect of skewness on power to detect bimodality. Samples were drawn from gamma distributions with increasing skewness, and the false-positive rate was found to be highly sensitive to skewness. A gamma distribution with shape parameter 7 and scale parameter 1 resulted in as many as 36% of datasets exhibiting evidence for bimodality, whereas a more skewed gamma(2,1) distribution produces nearly 90% false positives. That is to say, skewed distributions are much more likely to provide evidence for bimodal transcript abundance than are symmetric ones. If the reason for the skew is biologic, then false positives are not a great concern because they still identify potential departures from uniformity that may be due to allelic differences.

However, statistical analysis of microarray data is based on the assumption of underlying normal distributions, and investigators typically take steps to remove skewness [28]. Logarithmic transformation is one such step, but more aggressive procedures such as Box-Cox transformations [29] and quantile normalization [30] explicitly transform the data to approximate a standard normal distribution as far as possible. The implications are discussed below.

Another common data transformation is use of the loess procedure to reduce the tendency for ratios of measurements of two dyes on a single array to be correlated with their intensity, due to differential labeling or degradation of the two dyes [31]. This procedure is particularly important for reference sample designs in which the treatments and references are labeled with different dyes. In dye-flip experiments dye effects will tend to cancel out, but the loess transformation should reduce the within-sample variance, often increasing power. It may not improve the accuracy of estimation of sample means, and under some circumstances loess transformation markedly reduces the detection rate of differential expression [32]. This is the case here, because the right-hand side of Table 1 shows a 20% decrease in the rate of detection of multimodal transcription, after loess transformation. Only 50% of the NC multiple mode assignments (and just 32% of the CA) agreed between the raw and loess analyses. Although these cases allow some confidence in the interpretation, they also highlight sensitivity to data analysis approaches.

### Transcriptional bimodality in CEPH lymphoblast cell lines

**Figure 6:**
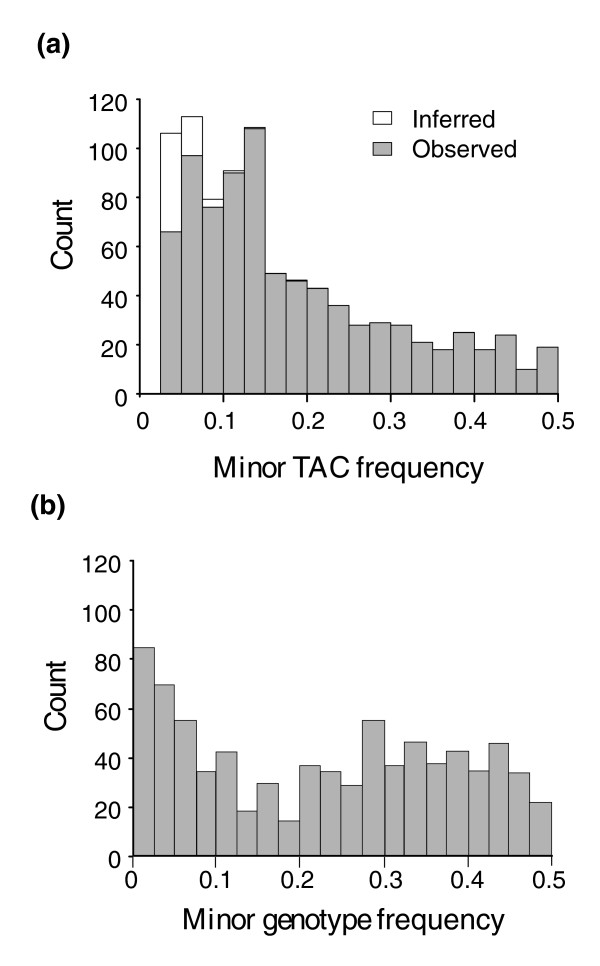
Transcript abundance classes in human cell lines. **(a) **The frequency distribution of transcript abundance classes (TACs) in the Centre d'Etude du Polymorphisme Humain data for 831 bimodally expressed genes. Open bars show the detected frequency of transcripts in each bin, and solid bars the reconstituted distribution adjusted for the false-negative detection rate for each bin. **(b) **The distribution of genotype frequencies for single nucleotide polymorphism (SNP) within 100 kilobases of each of the 831 transcripts that shows the strongest association with transcript abundance. Genotype is represented as the lesser of the common homozygote class or the sum of the heterozygotes and less common homozygote classes. This distribution is therefore right-shifted relative to the minor allele frequency distribution (and selection of SNPs with strong association statistics also biases the analysis toward common SNPs).

To determine whether the relatively high frequency of less common minor TACs is unique to *Drosophila*, a similar analysis of transcript abundance in lymphoblast cell lines derived from 40 grandparents in the CEPH pedigrees [12,13] was performed. As shown in Figure 6a, the same general left-shift in the TAC frequency distribution is observed in the 831 bimodally expressed genes. Unlike the *Drosophila *inbred lines, the human cell lines segregate three genotypes at most loci, and most of the minor homozygote classes are likely to be seen in fewer than 5% of the lines. Consequently, bimodality might be expected to be more commonly associated with the comparison of heterozygotes with the major homozygote class. The predicted distribution of these genotype groupings, given the observed allele frequencies for the SNP that shows the strongest association with expression in each of the bimodally expressed genes, is shown in the histogram in Figure 6b. Once again, there is some correspondence between the shape of the TAC frequency distribution and that of the expected genotype distribution. Note that 50 more transcripts exhibit multimodality, but the third and fourth transcript abundance classes are almost always rare, and power to detect these types of sample is low.

The availability of a dense SNP map for the CEPH samples [33] allowed us to scan for association between SNPs and transcript abundance in the bimodally expressed genes. Surprisingly, there is little overlap between our list of bimodally expressed genes and the transcripts associated with strong *cis*-regulatory polymorphisms reported by others [13,34]. This clearly indicates that only a fraction of *cis*-regulatory polymorphisms result in bimodal distributions of transcript abundance.

**Figure 7:**
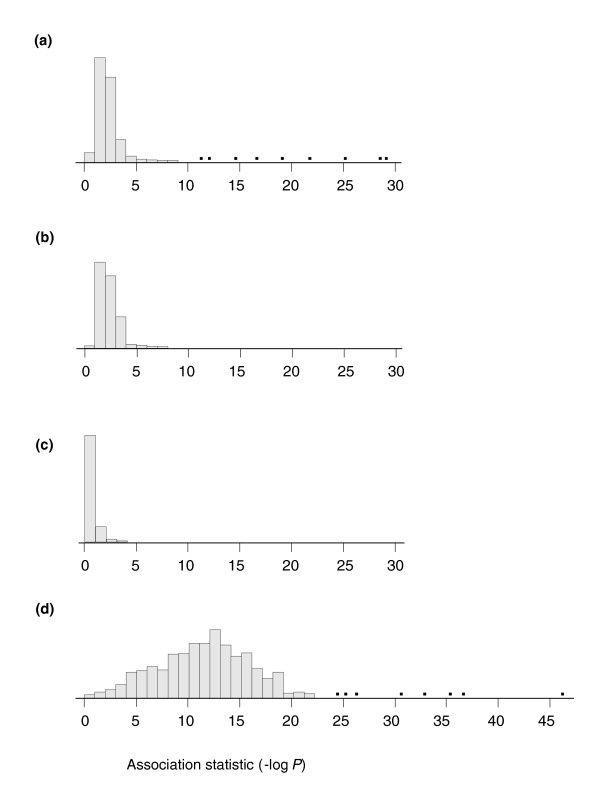
Strength of association between *cis*-SNPs and transcript abundance. Frequency histograms in bins of increasing order of magnitude of significance, with number of genes indicated on the y-axis. **(a) **The distribution of significance measures (negative logarithm of the *P *value) for the most strongly associated single nucleotide polymorphism (SNP) within 100 kilobases of each of the 818 bimodally expressed transcripts. **(b) **The same distribution for SNPs linked to a set of 835 randomly selected transcripts. Note the excess of outliers in the bimodal sample. **(c) **The distribution of strongest associations for a typical permutation of SNPs against unlinked transcripts, clearly showing much reduced significance relative to those observed for linked SNPs. **(d) **The 'best possible' distribution of associations, assuming that a single SNP explains all of the observed bimodality of each transcript. Single dots in panels A and D represent outlier significance values.

On the other hand, comparison with the distribution of *cis *associations in the set of bimodal TACs implies some enrichment for locally acting regulatory polymorphisms. Figure 7 shows the observed quantile distributions of the strongest association statistic for each gene in (panel a) our sample of 818 bimodal transcripts, (panel b) a random sample of 838 transcripts, (panel c) a random permutation of genotypes against transcripts, and (panel d) the best possible TAC associations, assuming that each TAC is due to a single genotype class (see Materials and methods, below). The distributions in panels a and b are similar overall, expect for the long tail encompassing the top 2.5% of the bimodal TAC sample, identifying 20 genes for which the two TACs are largely explained by single *cis*-acting SNPs. By contrast with panel c, random sets of genes are also heavily enriched for *cis*-acting SNPs, whose effects are not strong enough to exceed an experiment-wide significance threshold, but nevertheless strongly suggest that the majority of genes are regulated in part by *cis*-SNPs that have stronger associations than are observed if genotypes are randomly matched to transcript frequencies. Figure 7d indicates that most of the detected associations only explain a small portion of the bimodality of transcript abundance, because the association statistics are in general much smaller than would be observed if there were tight correspondence between genotype and transcript abundance.

Evidence for involvement of *trans*-acting factors in regulating gene expression would be found in a higher than expected incidence of sharing of TACs across lines. Because it is not trivial to estimate the expected proportion of sharing for abundance classes of hundreds of transcripts at different frequencies, we focused on rare TACs (those observed in just two or three lines). As described in Additional data file 1, in general these rare TACs are dispersed randomly across most of the lines. However, in all three datasets (the NC and CA samples of flies and the CEPH cell lines) a handful of individuals exhibit an excess of rare TACs, as well as a significant tendency for such rare abundance classes to be shared. This may be indicative of co-regulation by a *trans*-acting factor, although the phenomenon might also be due to an uncharacterized technical artifact.

## Discussion

What is the distribution of transcriptional variance within and among populations, and why does it matter? The short answers are that we have very little idea, but that because transcription provides a link between genotype and phenotype, an understanding of the complex mapping of these two attributes requires knowledge of the relationship between genetic and gene expression variation. We have good tools for quantifying genotypic variation, and an established population genetic theory describing the expected distribution of polymorphism. No such tools or theory yet exist to help us to evaluate the contributions of drift, mutation, selection, and admixture to shaping variation in gene expression. Consequently, there is a large gap in our appreciation of the molecular basis of phenotypic evolution and the population structure of disease susceptibility.

Mixture modeling appears to be a useful tool for detecting transcripts that are variable in abundance within populations, although its utility for comparing distributions between populations is yet to be established. Unfortunately, a large batch effect confounded the comparison of the two populations, and this limited our ability to apply an alternative analytical approach, namely Q_ST _analysis [6,35]. Q_ST _is a quantitative analog of the inbreeding coefficient, F_ST_, which is commonly used to quantify divergence between populations based on allele frequencies [36]. Simultaneous measurement of *Q*_ST _and *F*_ST _with genotypic markers at the same locus has the potential to facilitate tests of selection. Two recent studies of mutation accumulation in nematodes and *Drosophila *[37,38] both imply that stabilizing selection is pervasive at the transcriptional level, because natural isolates appear to harbor less variation than would be predicted based on the rate of genetic divergence of laboratory lines. Consequently, simultaneously high Q_ST _and F_ST _values may indicate adaptive divergence caused by linked regulatory polymorphism. Discordance between the parameters could have numerous causes, including the role played by *trans*-acting polymorphism in transcriptional variation and the possibility that major effect haplotypes accentuate population differences in transcript abundance.

Is there evidence for divergence between the NC and CA samples of flies? Batch effects may influence any large-scale microarray experiment, and so it is preferable that two populations be measured at the same time. Reduced costs and increased availability of single channel platforms for model organisms will soon allow parallel measurement of thousands of samples, which should facilitate comparisons based on mean transcript abundance. Here, though, we have focused on measures based on the variance and distribution of abundance among lines. Because only 14% of bimodal NC transcripts are also bimodal in CA, it might be argued that divergence in the frequency of polymorphisms that contribute to the bimodality is common. However, 50 lines per sample is at the lower limit of power, particularly given that half of the cases are due to relatively rare minor TACs. The examples presented in Figure 3 demonstrate that the proportions of the two major TACs are preserved between the populations at least in some cases. *Drosophila melanogaster *has traditionally been regarded as a panmictic species, with most of the variation shared among populations (for comparison, see [39]). However, as sequences replace allozyme studies, it has become apparent that, as in humans, a few percent of the variation does exhibit population structure, and that rare private alleles are not uncommon [40,41]. Although the bulk of the transcriptome is undifferentiated between the two North American populations, it is likely that further studies will confirm subtle divergence for a subset of transcripts.

Statistical analysis is often regarded as an objective enterprise that is guaranteed to arrive at an unambiguous test of a hypothesis. However, philosophical issues have impacts on interpretation of functional genomic studies. Frequentist and Bayesian analyses are not always in agreement [42,43], whereas disputes also arise over the adoption of false-positive or false-discovery rates as the most appropriate approach to identification of candidate genes [44,45]. This study highlights another analytical issue, namely the degree of aggressiveness in data normalization, which actually gives rise to an epistemological problem. It is obviously good practice to remove sources of bias and noise in data as much as possible without disrupting the biologic content, before model fitting. A major source of bias in gene expression profiles, whether contrasting population samples or tissues and treatments, is skew away from normality. Recognizing that skewness will tend toward false attribution of multiple mixtures, it could be argued that quantile normalization or some equivalent procedure should be used to force the data toward symmetric normal distributions. The trouble with doing this is that it will tend to 'throw the baby out with the bathwater'; skew is actually an expected property of distributions that arise from mixtures of two or more underlying distributions. We thus arrive at a biologic uncertainty principle, insofar as the process of measurement may destroy the signal.

What is the cause of the bias toward low frequencies of minor transcript abundance classes? For genotypes, it is well known that observed distributions approximate well to expectations under mutation-drift equilibrium, implying that a large proportion of molecular polymorphism segregates at or near neutrality. The loose correspondence between genotype and TAC frequency distributions is consistent with an extension of this principle, namely that a large proportion of bimodal transcriptional variation is due to the effect of nearly neutral *cis*-acting polymorphisms. However, the error associated with detection and measurement of TACs precludes rigorous testing of this hypothesis, and evidence was presented that *cis*-acting effects only account for a small proportion of bimodality. Across the 818 genes in the CEPH data, and for the one *Drosophila *gene examined closely, SNPs linked to the transcripts do not define transcript abundance classes. Consistent with results from expression QTL experiments [46], these results provide further evidence that expression tends to be regulated by a complex mixture of *cis*-acting and *trans*-acting factors. The shape of the TAC distributions in our data could be explained either by combination of the effects of relatively rare alleles or by low frequency combinations of independently segregating common alleles.

## Conclusion

Population profiling complements expression QTL analysis [7,8] as an approach to identification of candidate QTLs, because crosses derived from just two lines will tend to miss rare alleles in a population. Even in a common disease/common variant model of disease susceptibility, differential expression may only be expected in a small percentage of individuals. Mixture modeling appears to lose efficiency for detecting minor TACs that constitute less than 10% of the sample, but it nevertheless provides evidence for distinct abundance classes for between 5% and 10% of transcripts. The enrichment for transcripts regulated in *cis *is clearly only modest, with just 20 transcripts in the CEPH dataset showing more highly significant associations with linked SNPs than those observed for a random set of genes of the same size. On the other hand, both the bimodal and randomly chosen samples of genes exhibit an excess of significant associations relative to SNPs chosen from unlinked genes, providing further evidence for the pervasive contribution of *cis*-regulatory polymorphism to regulation of gene expression. The collection of strongly bimodally expressed transcripts from population sampling provides a sample of candidate genes that can be assessed for regulation of quantitative traits in targeted crosses or carefully chosen pedigrees.

## Materials and methods

### Experimental design

This experiment was conceived initially to evaluate transcriptional variation for response to nicotine in *D. melanogaster *[14]. Briefly, 58 isofemale lines from NC and 50 from CA were inbred by between 15 and 50 generations of sib-pair mating. These were chosen from a large sample of nearly isogenic lines that were described previously [41]. Residual heterozygosity is typically on the order of 0.1. The flies were reared in vials with standard cornmeal (control). Nicotine treatment was administered by transfer for 8 hours to standard cornmeal supplemented with a trace of nicotine. Because the majority of transcripts are relatively unaffected by the drug treatment, we simply averaged the two control and two nicotine treatments to obtain the line means. Analysis by drug treatment separately yields similar results.

RNA was extracted from flash-frozen heads of 50 adult 3-day to 5-day females for each line. For each population, collection was spread over 2 months, and the collections were performed 6 months apart. Hybridizations were performed with a pool of two separate linear amplification labeling reactions with either dye, Cy5 or Cy3. A randomized loop design ensured that each microarray contrasted a control to a nicotine sample, each drawn from a different line from the NC or CA sample (see Additional data file 2). There are a total of 216 microarrays, such that each of the 108 lines is represented by two control and two nicotine treatments with one dye-flip each.

The cDNA arrays were printed on glass slides at the NC State University Genome Research Laboratory using clones supplied by the Berkeley Drosophila Genome Project. A total of 223 of the 4,608 spots on the array lack polymerase chain reaction products or were missing for other technical reasons. Another group of spots consistently showed low intensity comparable to the empty spots, presumably because they represent genes that are not expressed in adult female heads. They were also excluded from analysis so as not to skew the distribution of effects across the whole array, resulting in 4,212 spots in our final analysis.

### Data transformation

The expression level for each transcript was estimated after first transforming the raw intensity measures with a log base-2 function. A global normalization for all arrays was performed using a linear mixed model of the following form:

y_ijk _= μ + D_i _+ T_j _+ (D × T)_ij _+ A_k _+ ε_ijk_

Where y is the log 2 intensity, μ is the overall mean, D_i _is the ith dye effect, T_j _is the jth treatment effect, and (D × T)_ij _is their interaction effect. Each of these terms is specified as a fixed effect, whereas A_k _is the random effect of the kth array and is assumed to be normally distributed with a mean of zero and variance σ_a_^2^.

Subsequently, a second gene-specific model was fit to estimate the true line effect for each gene effect, as follows:

rfi_ijkl _= G + (GD)_i _+ (GT)_j _+ (GD × GT)_ij _+ (GA)_k _+ (GL)_l _+ ε_ijkl_

where rfi is the relative fluorescence intensity, namely the residual from the global normalization; G is the overall mean; (GD)_i _is the ith gene-specific dye effect; (GT)_j _is the jth gene-specific treatment effect; (GD × GT)_ij _is their interaction term; and (GL)_l _is the gene-specific line effect for the lth line. All of these main effects are specified as fixed effects, as is the pair-wise interaction term. (GA)_k _is the gene-specific random effect for array variation. Least squares means of the gene-specific line effect are derived to represent the expression level of each line. It should be noted that use of a randomized loop will tend to reduce the total among line variance, because if two lines with high (or low) abundance happen to be hybridized on the same array, then part of the differential expression will be absorbed into the estimate of the array effect, (GA)_k_.

### Mixture modeling

A simple one-dimensional multi-mixture model was constructed for each gene based on the least squares means of the line effect from the mixed model described above. Each model assumes a weighted sum of normal distributions, which are called mixtures:

$$\text{f}(\text{l})=\sum_{\text{i}=1}^{\text{m}}{\text{w}}_{\text{i}}\times \text{N}(\mu_{\text{i}},\sigma_{\text{i}}^{\text{2}})$$

Where f(l) is the distribution of least squares mean line effects, and w is the weighting of each normal distribution, namely the proportion of samples classified into that component. The sum of the w_i_s should be equal to 1. The μ_i _and σ_i_^2 ^are the mean and variance of the normal distribution, and m is the number of mixtures fitted. We used the EMclust EM algorithm within a package in R called Mclust for the modeling [17]. This package also allows choice of the number of mixtures that provides the optimal fit to the data, based on a Bayesian Information Criterion that accounts for the difference in degrees of freedom associated with each model. The maximum number of mixtures was restricted to 5.

The allelic frequency spectrum in Figure 4 was derived as described in Ewens' 1972 paper [19] as following: f(x) = θ x^-1^(1 - x)^θ-1^, where θ = 4Nμ. In other words, f(x)δ × is the probability that an allele in the population will be in the frequency range (x, x + δ) for small δ. Based on the generally accepted range θ for *Drosophila*, we choose 0.05, 0.1, and 0.2 for demonstration. For θ → 0, f(x) is approximately a symmetric function with respect to 0.5, so the minor allele frequency can be formulated as twice the function f with the range of (0,0.5).

### Transcriptional bimodality

Genes were classified as bimodally expressed when the optimal number of mixtures fitted by Mclust was m = 2. For each such gene, the frequency of the minor TAC was defined as the smaller of the weights w_1 _and w_2 _in the estimated two-component mixture model w_1 _× N(μ_1_,σ_1_^2^) + w_2 _× N(μ_2_, σ_2_^2^). Because we expected that the parameters of the underlying mixture distribution would influence the resolution of bimodality, we anticipated an ascertainment bias in the empirical distribution of TAC frequencies; to recover the latent TAC frequency distribution, we sought a simulation-based estimate of this bias. For each gene classified as bimodally expressed, we used the estimated two-component mixture model to generate 10,000 samples of 58 observations. We then used Mclust on each of the 10,000 samples, recording the proportion of successful mixture resolutions. This approach, reminiscent of the parametric bootstrap, yielded a gene-specific estimate β of the power to detect bimodality; we attributed the false-negative rate 1 - β to a latent class of bimodally expressed genes that went undetected by Mclust. In other words, a bimodally expressed gene with a power estimate of β stands a probability of 1 - β of going undetected, and to correct for this ascertainment bias we counted the gene 1/β times. In particular, by weighting the minor TAC frequency of each gene by the reciprocal of its simulated power, we obtained a TAC frequency distribution that has been disentangled from the discovery process.

### Tests of association in the CEPH data

In order to evaluate the level of association between *cis*-SNPs and bimodality, we extracted from the HapMap database all SNPs within 100 kb of each of the 881 multimodal transcripts from the mixture modeling. Sixty-three of these transcripts either had more than two modes or are not annotated sufficiently well to identify linked SNPs, resulting in a final set of 818 genes. A random sample of 881 other genes resulted in 838 genes with well annotated linked SNPs within 100 kb. We then performed a *t*-test of the difference in estimated transcript abundance between the major homozygote class and the joint set of heterozygotes and minor homozygotes, and simply report the distribution of strongest associations for each SNP and transcript. Neither a tagging strategy nor a minor allele frequency cutoff was employed, and nor was a multiple correction factor used. Either of these would certainly be important were we to make any claims about a specific association, but Figure 7 deals only with the distribution of all the statistics obtained as described, and the conclusions would not be affected greatly by alternate analyses.

## Additional data files

The following additional data are available with the online version of this paper. Additional data file 1 summarizes the analysis of the distribution of rare TACs among lines within each of the three experimental datasets. Additional data file 2 illustrates the experimental design.

All expression data are available from the MIAME compliant public repository at ArrayExpress [47], expression profile number E-TABM-109, and from our laboratory supplementary information site [48], which provides various other support files including SAS scripts, data analysis summaries, and array annotation files.

## Supplementary Material

Additional data file 1 This document summarizes the analysis of the distribution of rare TACs among lines within each of the three experimental datasets. Click here for file

Additional data file 2 The experimental design is illustrated. Each line is represented by four measurements: two involving control samples and two nicotine-treated samples. These were obtained from four microarrays, with a balance of Cy3 and Cy5 dyes, and a randomized loop. For example, line 2 was hybridized as control Cy3 to line 5 nicotine Cy5 on one array, and control Cy5 to line 1. Two different loops were generated, one for each population. Click here for file

## References

[B1] Ewens W (2000). A hundred years of population genetics theory.. J Epidemiol Biostat.

[B2] Ohta T, Gillespie JH (1996). Development of neutral and nearly neutral theories.. Theor Popul Biol.

[B3] Orr HA (2005). The genetic theory of adaptation: a brief history.. Nat Rev Genet.

[B4] Monks SA, Leonardson A, Zhu H, Cundiff P, Pietrusiak P, Edwards S, Phillips JW, Sachs A, Schadt EE (2004). Genetic inheritance of gene expression in human cell lines.. Am J Hum Genet.

[B5] Cheung VG, Conlin LK, Weber TM, Arcaro M, Jen K-Y, Morley M, Spielman RS (2003). Natural variation in human gene expression assessed in lymphoblastoid cells.. Nat Genet.

[B6] Gibson G, Weir B (2005). The quantitative genetics of transcription.. Trends Genet.

[B7] Stamatoyannopoulos JA (2004). The genomics of gene expression.. Genomics.

[B8] de Koning DJ, Haley CS (2005). Genetical genomics in humans and model organisms.. Trends Genet.

[B9] Rockman MV, Wray GA (2002). raw material for *cis *Abundant -regulatory evolution in humans.. Mol Biol Evol.

[B10] Dermitzakis ET, Clark AG (2002). Evolution of transcription factor binding sites in Mammalian gene regulatory regions: conservation and turnover.. Mol Biol Evol.

[B11] Wittkopp PJ, Haerum BK, Clark AG (2004). Evolutionary changes in *cis *and *trans *gene regulation.. Nature.

[B12] Morley M, Molony C, Weber T, Devlin J, Ewens WK, Spielman RS, Cheung VG (2004). Genetic analysis of genome-wide variation in human gene expression.. Nature.

[B13] Cheung VG, Spielman RS, Ewens KG, Weber TM, Morley M, Burdick JT (2005). Mapping determinants of human gene expression by regional and genome-wide association.. Nature.

[B14] Fondation Jean Dausset - CEPH. http://www.cephb.fr.

[B15] Passador-Gurgel G, Hsieh WP, Hunt P, Deighton N, Gibson G (2007). Quantitative trait transcripts for nicotine resistance in *Drosophila melanogaster*.. Nat Genet.

[B16] Churchill GA (2002). of experimental design for cDNA microarrays.. Nat Genet.

[B17] Stapleton M, Liao G, Brokstein P, Hong L, Carninci P, Shiraki T, Hayashizaki Y, Champe M, Pacleb J, Wan K (2002). The *Drosophila *gene collection: identification of putative full-length cDNAs for 70% of *D. melanogaster *genes.. Genome Res.

[B18] Fare TL, Coffey EM, Dai H, He YD, Kessler DA, Kilian KA, Koch JE, LeProust E, Marton MJ, Meyer MR (2003). Effects of atmospheric ozone on microarray data quality.. Anal Chem.

[B19] Pearson K (1894). Contributions to the mathematical theory of evolution.. Phil Trans Roy Soc A.

[B20] Fraley C, Raftery AE (1999). Mclust: software for model-based cluster analysis.. J Classification.

[B21] Yeung KY, Fraley C, Murua A, Raftery AE, Ruzzo WL (2001). Model-based clustering and data transformations for gene expression data.. Bioinformatics.

[B22] Ewens W (1972). The sampling theory of selectively neutral alleles.. Theor Popul Biol.

[B23] Griffiths RC, Lessard S (2005). Ewens' sampling formula and related formulae: combinatorial proofs, extensions to variable population size and applications to ages of alleles.. Theor Popul Biol.

[B24] Aquadro CF, Bauer DuMont V, Reed FA (2001). Genome-wide variation in the human and fruitfly: a comparison.. Curr Opin Genet Dev.

[B25] Palsson A, Gibson G (2004). Association between nucleotide variation in *Egfr *and wing shape in *Drosophila melanogaster*.. Genetics.

[B26] Nikoh N, Duty A, Gibson G (2004). Effects of population structure and sex on association between serotonin receptors and *Drosophila *heart rate.. Genetics.

[B27] Macdonald SJ, Pastinen T, Long AD (2005). The effect of polymorphisms in the enhancer of split gene complex on bristle number variation in a large wild-caught cohort of *Drosophila melanogaster*.. Genetics.

[B28] Quackenbush J (2002). Microarray data normalization and transformation.. Nat Genet.

[B29] Durbin B, Rocke DM (2003). of transformation parameters for microarray data.. Bioinformatics.

[B30] Bolstad BM, Irizarry RA, Astrand M, Speed TP (2003). A comparison of normalization methods for high density oligonucleotide array data based on variance and bias.. Bioinformatics.

[B31] Yang YH, Dudoit S, Luu P, Lin DM, Peng V, Ngai J, Speed TP (2002). Normalization for cDNA microarray data: a robust composite method addressing single and multiple slide systematic variation.. Nucl Acids Res.

[B32] Dabney AR, Storey JD (2007). Normalization of two-channel microarrays accounting for experimental design and intensity-dependent relationships.. Genome Biol.

[B33] International HapMap Consortium (2005). A haplotype map of the human genome.. Nature.

[B34] Stranger BE, Forrest MS, Clark AG, Minichiello MJ, Deutsch S, Lyle R, Hunt S, Kahl B, Antonarakis SE, Tavare S (2005). Genome-wide associations of gene expression variation in humans.. PLoS Genet.

[B35] Ritland K (2000). Marker-inferred relatedness as a tool for detecting heritability in nature.. Mol Ecol.

[B36] Weir BS, Hill WG (2002). Estimating F-statistics.. Annu Rev Genet.

[B37] Denver DR, Morris K, Streelman JT, Kim SK, Lynch M, Thomas WK (2005). The transcriptional consequences of mutation and natural selection in *Caenorhabditis elegans*.. Nat Genet.

[B38] Rifkin SA, Houle D, Kim J, White KP (2005). A mutation accumulation assay reveals a broad capacity for rapid evolution of gene expression.. Nature.

[B39] Dieringer D, Nolte V, Schlotterer C (2005). Population structure in African *Drosophila melanogaster *revealed by microsatellite analysis.. Mol Ecol.

[B40] Spicer GS, Fleming JE (1991). Genetic differentiation of *Drosophila melanogaster *populations as assessed by two-dimensional electrophoresis.. Biochem Genet.

[B41] Palsson A, Rouse A, Riley-Berger R, Dworkin I, Gibson G (2004). Nucleotide variation in the *Egfr *locus of *Drosophila melanogaster*.. Genetics.

[B42] Efron B, Tibshirani R (2002). Empirical bayes methods and false discovery rates for microarrays.. Genet Epidemiol.

[B43] Ranz JM, Namgyal K, Gibson G, Hartl DL (2004). Anomalies in the expression profile of interspecific hybrids of *Drosophila melanogaster *and *Drosophila simulans*.. Genome Res.

[B44] Qian HR, Huang S (2005). Comparison of false discovery rate methods in identifying genes with differential expression.. Genomics.

[B45] Broberg P (2005). A comparative review of estimates of the proportion unchanged genes and the false discovery rate.. BMC Bioinformatics.

[B46] Brem RB, Kruglyak L (2005). The landscape of genetic complexity across 5,700 gene expression traits in yeast.. Proc Natl Acad Sci USA.

[B47] ArrayExpress. http://www.ebi.ac.uk/arrayexpress.

[B48] Supplementary Information for Hsieh, Passador-Gurgel, Stone and Gibson. http://statgen.n csu.edu/ggibson/SupplInfo/SupplInfo12.htm.

