# Supplementary material for: Mixture modeling of transcript abundance classes in natural populations
Source: Genome Biol. 2007 Jun 4;8(6):R98. doi: 10.1186/gb-2007-8-6-r98 (PMC2394757; doi:10.1186/gb-2007-8-6-r98)
Supplement: Additional data file 1 — This document summarizes the analysis of the distribution of rare TACs among lines within each of the three experimental datasets. [file gb-2007-8-6-r98-S1.doc]

**1. North Carolina Sample**

In the NC fly lines, we find that 304 genes have exactly two TACs. Among these 304 genes, 82 have rare TACs, defined here as TACs with observed frequencies less than or equal to 3/58. The following two-way clustering of TACs shows their distribution among lines. Each column represents a line and each row represents a gene, with rare TACs represented by blue blocks in the matrix. Gray vertical bars indicate lines devoid of minor TACs for all of the 82 genes.


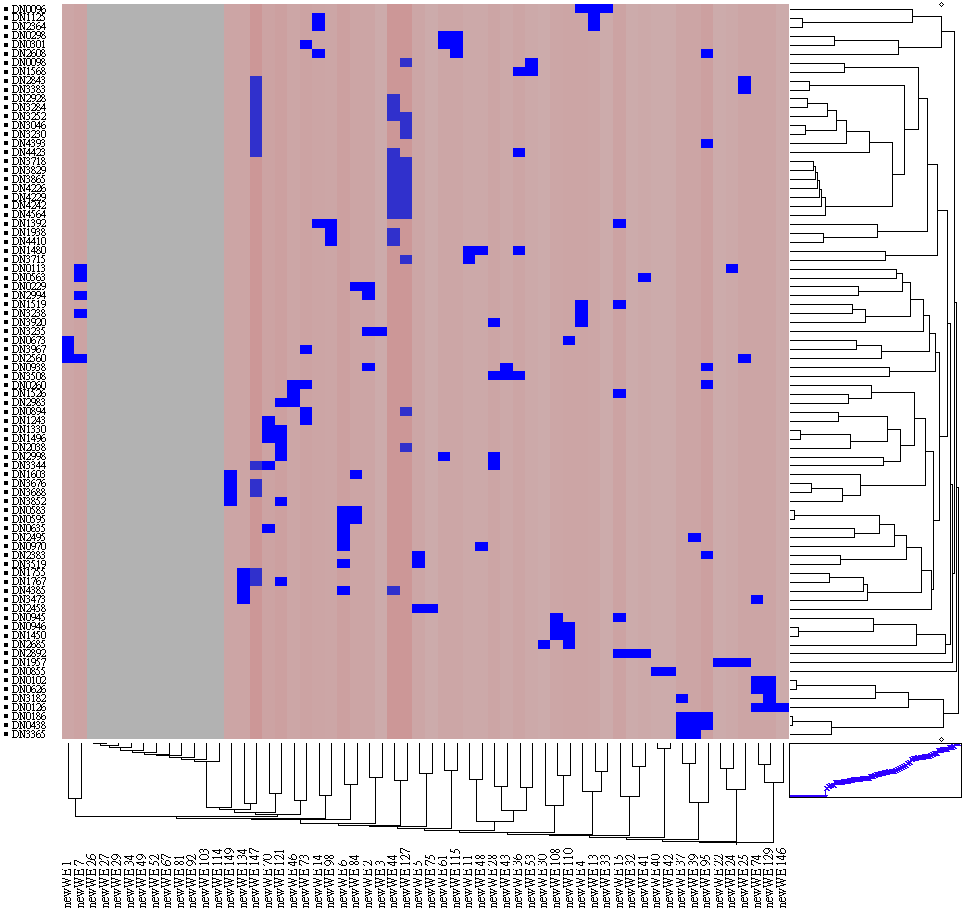


The distribution of rare TACs per line is shown on the next page. Most lines have only a few rare TACS, but lines 44 and 127 appear to share eight rare TACs, as well as having many more rare TACs overall (14) than the other lines (excepting line 147). The probability that the number of rare TACs shared between the two lines is greater than or equal to 8 is 1.593e-4, calculated as follows:

$$\begin{matrix} P(\#\text{rare TACs shared}=i) \\ =\frac{\left(\begin{matrix} 82 \\ 14 \end{matrix}\right)\left(\begin{matrix} 14 \\ i \end{matrix}\right)\left(\begin{matrix} 68 \\ 14-i \end{matrix}\right)}{\left(\begin{matrix} 82 \\ 14 \end{matrix}\right)\left(\begin{matrix} 82 \\ 14 \end{matrix}\right)} \end{matrix}$$

Due to the limitation of mixture model clustering, rare TACs of frequency 1/58 cannot be detected. Therefore, for each line, in computing the probability of a rare TAC at gene *i* (where *i* = 1….82) we only consider frequencies of 2/58 and 3/58. If the rare TAC frequency is 2/58 for all genes, the probability that any line has *x* rare TACs follows a binomial distribution BIN(82, 2/58) based on the assumption of independence among genes. See the expected (red) and observed (blue) distribution in the following left panel. The same plot for all rare TACs with frequency 3/58 is plotted on the right. In our case, 20 of the 82 genes have rare TACs frequency of 2/58 while the rest of them are of frequency 3/58. The expected distribution is between the two graphs. A simple chi-squared test implies that the observed distribution is significantly different from either of the two distributions, due to an excess number of observations for lines with only zero or one rare TAC. Aside from lines 44 and 127, which may be a technical artifact, the number of rare TACs shared between any two lines is no greater than expected by chance.


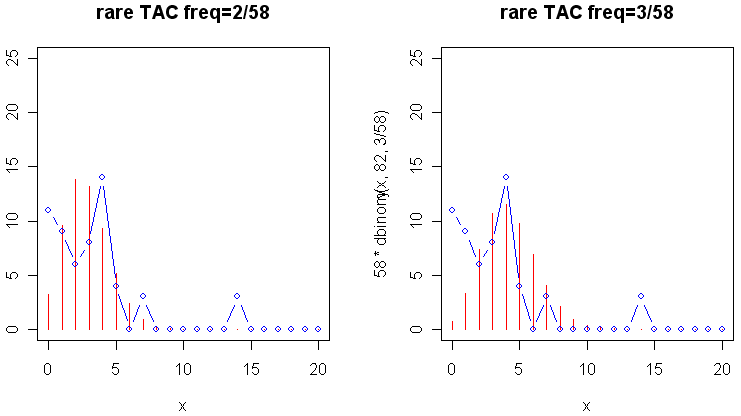


The number of rare TACs shared for any pair of lines is distributed as follows:

|  | With 0 rare TACs in common | With 1 rare TACs in common | With 2 rare TACs in common | With 3 rare TACs in common | With 4 rare TACs in common | With 8 rare TACs in common |
| --- | --- | --- | --- | --- | --- | --- |
| Pairs of lines | 1558 | 79 | 11 | 3 including (127,147) | 1  (44, 147) | 1  (44, 127) |

**2. California Sample**

In the Californian fly lines, we find that 369 genes have exactly two TACs. Among these 369 genes, 127 have rare TACs, defined here as TACs with an observed frequency less than or equal to 3/50. The following two-way clustering of TACs shows their distribution among lines. Each column represents a line and each row represents a gene, with rare TACs represented by blue blocks in the matrix. Gray vertical bars indicate lines devoid of minor TACs for all of the 127 genes.


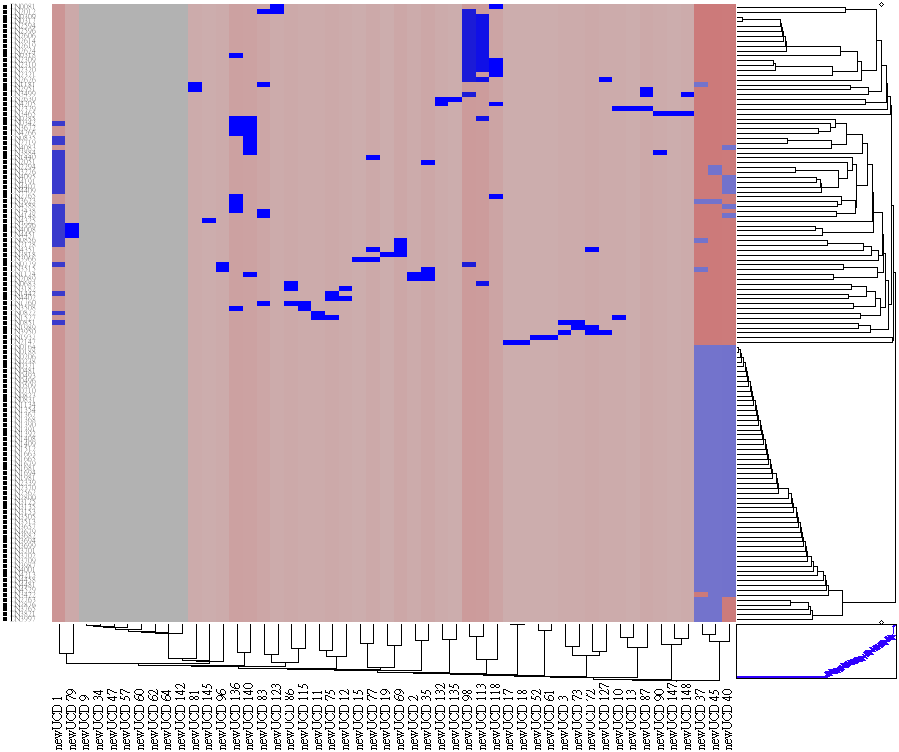


The distribution of rare TACs per line is shown on the next page. Most lines have only a few rare TACs, but lines 37, 40 and 45 at the far right seem to share more rare TACs than the other lines, and to have more rare TACs overall (59 or 60).

For this sample, the probability that any line has *x* rare TACs (less than or equal to 2/50) follows a binomial distribution BIN(127, 2/50) under the assumption of independence among genes. The expected (red) and observed (blue) distributions, truncated at 20 for illustration, again show an excess of lines with 0, 1 or 2 rare TACs.


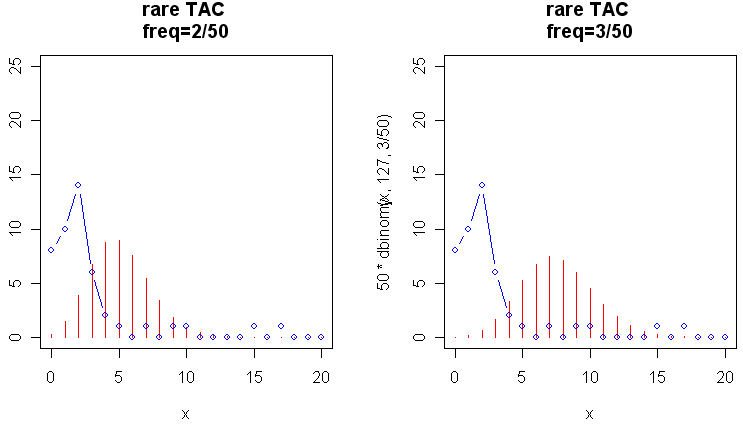


|  | With 0 rare TACs in common | With 1 rare TACs in common | With 2 rare TACs in common | With 3 rare TACs in common | With 4 rare TACs in common | With 13 rare TACs in common |
| --- | --- | --- | --- | --- | --- | --- |
| Pairs of lines | 1011 | 59 | 4 | 3 | 3 | 1 (CA 98, 113) |

If we remove line 37, 45 and 40 from the analysis, only 61 genes remain with rare TACs and the expected distribution is BIN(61, 2/47). The chi-squared test still supports non-independence across genes, but in this case is due to a few lines (98, 113, 118) sharing more than 10 rare TACs. This number is highly unlikely to occur by chance.


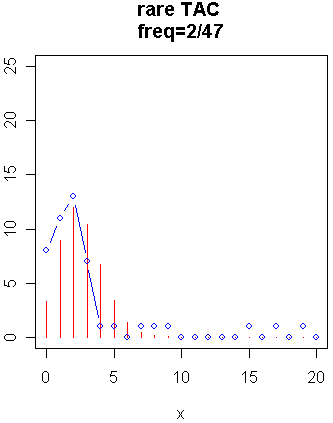


The behavior of the three special lines with 60 shared rare TACs is not due to spurious noise at low expression levels, since the rare TACs are all expressed at relatively high levels. Nor is there is no evidence for scratches or other artifacts on the array images, so the phenomenon appears to be biological. However, technical artifacts perhaps arising during cDNA labeling or preparation cannot be excluded.

**3. CEPH lines**

There are 881 multimodal genes in the CEPH data and 831 of them show exactly two components. Among these 831 genes, 132 have rare TACs, defined here as TACs with an observed frequency less than or equal to 3/58. The following two-way clustering of TACs shows their distribution among lines. Each column represents a line and each row represents a gene, with rare TACs represented by blue blocks in the matrix. Gray vertical bars indicate lines devoid of minor TACs for all of the 132 genes.


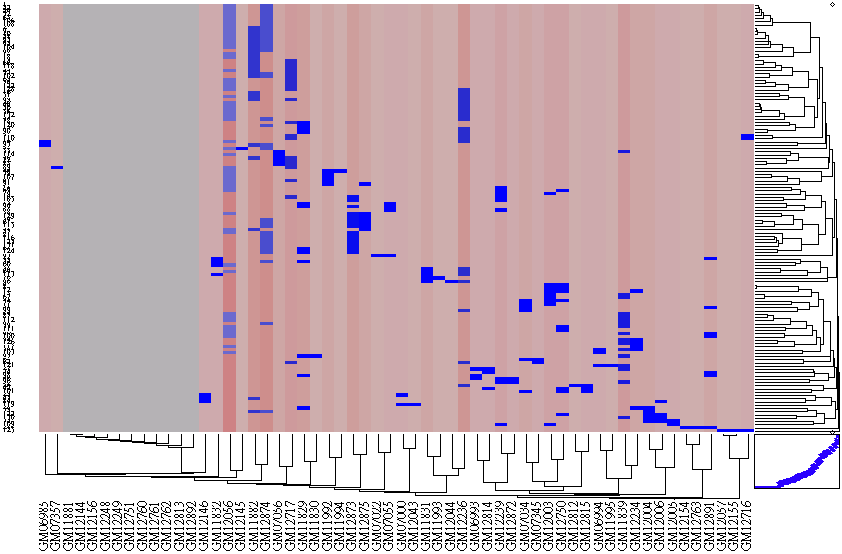


In this case, two cell lines, GM12056 and GM 12874, have considerably more rare TACs than any of the others, but again for the most part the rare TACs are scattered across all of the samples. Whether or not these lines are included in the analysis, there is a slight excess of lines sharing 10 or more rare TACs. The following plots of observed (blue) versus expected (red) distribtions are based on the 86 genes that remain after removing the two outlier lines. Two individuals, GM11882 and GM12717, share 8 rare TACs, while individual GM12717 has 18 rare TACs.

|  | 0 rare TACs | 1 rare TACs | 2 rare TACs | 3 rare TACs | 4 rare TACs | 5 rare TACs | 8 rare TACs |
| --- | --- | --- | --- | --- | --- | --- | --- |
| Pairs of lines | 1441 | 77 | 12 | 5 | 2 | 2 | 1 |


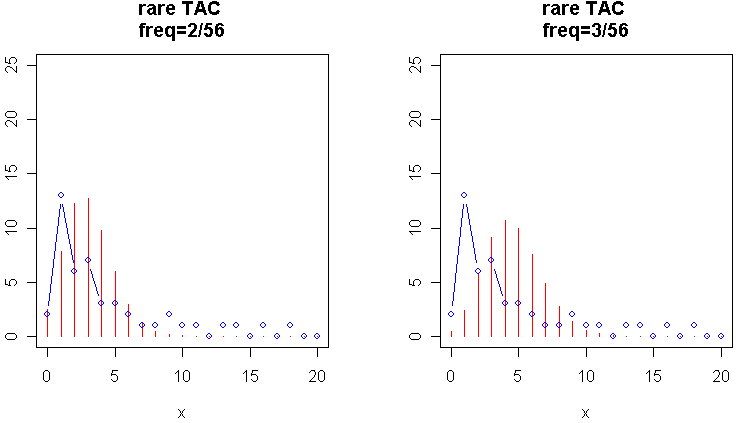


**Conclusions**

In all three samples, there are two or three lines that are distinct from the others in terms of both the number of rare TACs and the likelihood that they share the unusual expression levels of a subset of these transcripts. It is not clear whether this differentiation reflects a true biological phenomenon, or whether it might be attributed to the kinds of unspecified technical artifacts that plague microarray experiments.

For the most part, rare TACs are randomly distributed among lines. This result does not provide any information as to whether the differential expression is due to cis- or trans-acting variants. The excess sharing of rare TACs among a small number of lines is suggestive of the involvement of trans-acting differences that are either rare or only impact a few target genes.

However, in all three samples, the distribution of rare TACs is skewed slighty but significantly by a few lines with a larger than expected number of rare TACs. This excess probably also explains an excess of lines with zero or only a few observed rare TACs. Again, it is not clear whether the divergent lines represent technical artifacts or a true biological phenomenon.
