# Supplementary figures and images for: Mixture modeling of transcript abundance classes in natural populations
Source: Genome Biol. 2007 Jun 4;8(6):R98. doi: 10.1186/gb-2007-8-6-r98 (PMC2394757; doi:10.1186/gb-2007-8-6-r98)

**Supplementary Figure 1** Hsieh et al, 2006

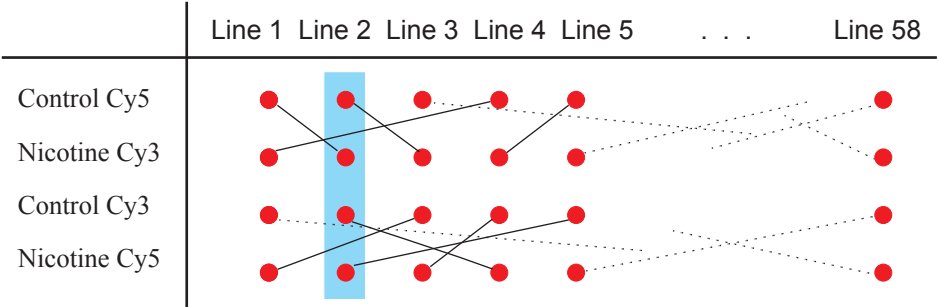

Supplement: Additional data file 2 — The experimental design is illustrated. Each line is represented by four measurements: two involving control samples and two nicotine-treated samples. These were obtained from four microarrays, with a balance of Cy3 and Cy5 dyes, and a randomized loop. For example, line 2 was hybridized as control Cy3 to line 5 nicotine Cy5 on one array, and control Cy5 to line 1. Two different loops were generated, one for each population. [file gb-2007-8-6-r98-S2.pdf]
